# From a Laboratory Exercise for Students to a Pioneering Biosensing Technology

**DOI:** 10.1007/s11468-013-9654-3

**Published:** 2014-01-16

**Authors:** Ingemar Lundström

**Affiliations:** Biosensors and Bioelectronics Centre, Department of Physics, Chemistry and Biology, Linköping University, 581 83 Linköping, Sweden

**Keywords:** Surface plasmon resonance, Historical overview, BIAcore, Computer screen photo-assisted techniques

## Abstract

Surface plasmon resonance (SPR) for biosensing was demonstrated 30 years ago. In the present contribution, its general background is described together with the necessary developments both in instrumentation and surface chemistry, leading to the final so-called BIAcore technology. The description is naturally colored by my personal opinion of the developments. SPR for the elucidation of organic mono- and multilayers introduced at the end of the 1970s formed the basis for the first biosensing demonstration of SPR in the beginning of the 1980s. It is pointed out how the need of an up-to-date laboratory exercise for the undergraduate students and the multidisciplinary environment at the Laboratory of Applied Physics at Linköping University led to this demonstration. The initial experiments are touched upon and the further developments at Pharmacia, which led to the BIAcore technology, are described in some details. Some of the present activities in Linköping related to optical biosensing with ubiquitous instrumentation are also described, including SPR detection with a computer screen and a web camera and most recently with a cellular phone.

## Introduction

It was demonstrated already in 1983 that the binding between non-labeled biomolecules could be detected with surface plasmon resonance (SPR) [1]. This demonstration initiated a commercial development, which led to a world-leading technology for biospecific interaction analysis, nowadays known as the BIAcore technology. A description of the early developments was published in Biosensors and Bioelectronics 1995 as a bioanalytical history report with the title “Biosensing with surface plasmon resonance—how it all started” [2]. The present contribution will also try to describe in some details the scientific environment, global and local, at the time of the original demonstration. The description is highly subjective and does not pretend to give full credit to all contributors to the background knowledge and to ideas which went into the development of the technology. References will mainly be given to work at the Department of Physics, Chemistry and Biology (earlier Department of Physics and Measurement Technology) instead of other original references in the field. The contribution is therefore not a proper review of the field of biomolecular interactions on surfaces but focuses on the work performed in Linköping. The multidisciplinary character of the Laboratory of Applied Physics was the consequence of the written program for the Chair of applied physics (which I got in 1978), where it was said “…applications of physics in chemistry, biology and medicine.” In the beginning of the 1980s, there were therefore engineers, physicists, chemists, and microbiologists working together, of which a handful was engaged in studies of protein adsorption and surface-related biological phenomena. The content of the paper is roughly as follows:

- Protein adsorption and protein interactions on surfaces studied with ellipsometry
- Experiments with surface plasmon resonance for the monitoring of thin (organic) layers on metals
- Gas sensing with surface plasmon resonance—a laboratory exercise
- Initial biosensing experiments with surface plasmon resonance
- Developments in making surface plasmon resonance into a pioneering biosensor technology
- Later activities in Linköping
- Conclusions

It should be pointed out already in the “Introduction” that although the original demonstration was made in the multidisciplinary environment at the Laboratory of Applied Physics, it was the contribution from Pharmacia which made SPR into a pioneering biosensor technology. The contribution was not only through their capital investment in the development but also due to several scientific and technical novelties put into it. The “Conclusions” will contain some personal reflections of why SPR ended up as the first leading biosensor technology in relation to other non-labeling possibilities like ellipsometry or mass changes detected with quartz crystal microbalances. It is observed that there will be no section specifically related to the physics of SPR. The physics will only be commented upon in connection to the choices made for the development of SPR into a useful biosensing technology.

As already stated, this contribution is not a proper review of surface plasmon resonance for biosensing. It lists mainly references to the work in Linköping and at Pharmacia Biosensor. It gives no credit to all the contemporary extremely interesting developments made at other places, and it contains nothing about the developments during the last 10 or 15 years of, for example, imaging SPR and localized SPR. These and other developments will no doubt be described in other parts of the present issue.

## Protein Adsorption and Protein Interactions on Surfaces Studied with Ellipsometry

It is fair to say that the scientific background to affinity-based biosensing without labeled molecules lies in the use of another surface-oriented optical technique, namely ellipsometry, where the polarization change of light upon reflection in a surface is measured. This is a very sensitive method which can be used, e.g., to detect submonolayer coverage of organic molecules on surfaces of different kinds (metal, insulator, semiconductor,…). It is used also to monitor the thickness and optical properties of thin layers in material science and semiconductor device technology. Several attempts of commercial developments of ellipsometry for biosensing purposes have been made both in Sweden and elsewhere, both before and after the demonstration of the SPR possibility. Although, to my knowledge, there are no developments as successful as the BIAcore and similar SPR-based technologies, ellipsometry has found numerous applications in biology. These are summarized in two reviews written by one of the first co-workers at the Laboratory of Applied Physics and one of the pioneers, regarding ellipsometry in Sweden [3, 4].

The use of ellipsometry for the study of the interaction of proteins with and on surfaces was pioneered by Vroman [5] and Rothen [6] at the end of the 1960s, including the binding between antigens and antibodies on the surface. One initial finding was that in a multi-protein solution, like plasma, there are both competition and replacement reactions on the surface. The latter, named the Vroman effect, results in that with time the stickier protein (generally the larger) molecules replace smaller molecules which have adsorbed on the surface first. The initial experiments indicated that ellipsometry could be used to study biological phenomena on surfaces, like surface-induced blood clotting and complement activation and how different surfaces would influence these phenomena. The use of ellipsometry for such studies was initiated in Sweden at Chalmers University of Technology [7, 8] and set up in Linköping in 1978 at the start of the Laboratory of Applied Physics. Some of the first applications of ellipsometry in the new laboratory were related to protein adsorption as a function of surface energy, model studies of surface-induced blood coagulation, and complement activation related to biomaterial surfaces [9–11]. The ellipsometer was used to measure both adsorption kinetics and isotherms and, through the use of specific antibodies also, the protein adsorption pattern under different conditions. The ellipsometer provided in a simple way the “optical” mass of organic molecules on the surface which yielded the information asked for. There was thus knowledge not only about surface-oriented optical evaluation of protein layers but also about antibody–antigen reactions on surfaces.

## Experiments with Surface Plasmon Resonance for the Monitoring of Thin (Organic) Layers on Metals

**Fig. 1:**
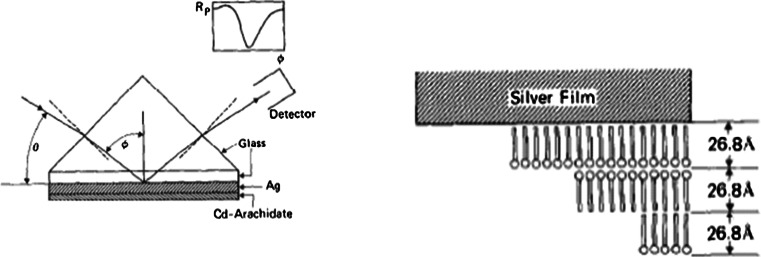
Experimental setup in the Kretschmann configuration and the schematic structure of the layer system (Reprinted from ref. [14] with permission from Elsevier)

**Fig. 2:**
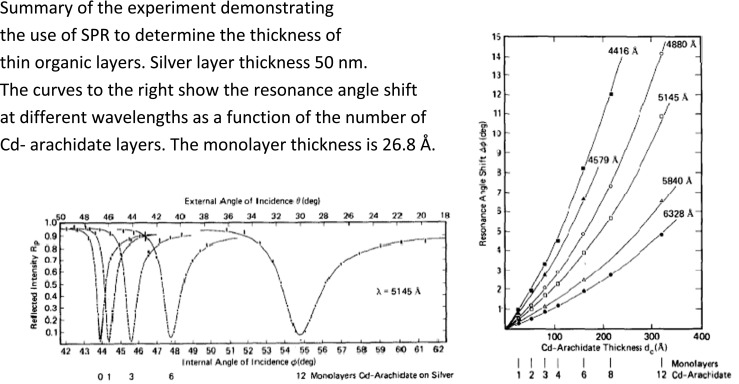
Experimental results obtained with the setup and film structure in Fig. 1 (Reprinted from ref. [14] with permission from Elsevier)

Erwin Kretschmann introduced in the beginning of the 1970s a practical method to excite surface plasmons in the surface of a metal, where light under total reflection conditions falls through a glass onto a thin metal film evaporated on the glass [12]. Kretschmann calculated the reflected light intensity through the use of Fresnel's reflection coefficients and was primarily interested in a method for the determination of the optical constants of metals. He demonstrated the possibility through an experiment on a layer of silver. Kretscmann showed, for example, experimentally that the optimal thickness of a silver film is about 60 nm. He also derived, however, an expression for the change in resonance condition caused by a thin layer on the metal surface. From a calculation assuming a silver layer thickness of 623 Å, wavelength 5,461 Å (*ε*_Ag_ = −12.03 + i 0.45) with a film of silver sulfide on its surface (*ε*_sulfide_ = 9 + i 3.5), he concluded that a 10-Å thick film would increase the resonance angle with about 0.4°. He gives as a conclusion that at the determination of optical constants, a layer on the metal surface can disturb the measurements. He also, however, points out that his method provides a good method for the determination of the optical constants and thickness of thin layers on the metal surface. The latter possibility was subsequently used to study organized mono- and multilayers of organic films on metal surfaces. Two papers from 1977 seem to be the first publications in which the Kretschmann configuration is used to experimentally detect thin organic layers on metals (gold and silver) [13, 14]. The experiment and results found in one of the publications are summarized in Figs. 1 and 2.

## Gas Sensing with Surface Plasmon Resonance—a Laboratory Exercise

The two previous sections give much of the scientific background, leading to the demonstration of SPR for biosensing purposes. It started, however, as a laboratory exercise for undergraduate students at our department. In the search for new exercises around 1980, one idea was to build a simple setup to demonstrate the surface plasmon resonance phenomenon. We had at that time developed a quartz microbalance sensor for anesthetic gasses (halogenated hydrocarbons) based on a silicone oil as the sensing layer [15]. It was reasoned that the refractive index changes occurring in the silicone oil should give an appreciable shift of the surface plasmon resonance angle and could serve the purpose not only as an interesting exercise for the students but also give the possibility of a new gas-sensing technology. The study showed that SPR performed quite well compared to a commercial instrument based on our quartz crystal microbalance sensor [16]. This experiment was to our knowledge the first demonstration of gas sensing with SPR. Actually, refs. [15] and [16] were published in the same issue of *Sensors and Actuators*. The anesthetic gas monitor based on a quartz crystal microbalance was, however, introduced at a conference 2 years earlier (1980) [17].

## Initial Biosensing Experiments with Surface Plasmon Resonance

The encouraging gas sensing results, discussions with and suggestions from microbiologists at the Laboratory of Applied Physics, led us to try immune sensing with SPR, which resulted in a now “classical” paper [1]. The initial experiments were made with a silver film evaporated on a microscope slide, which was used as one wall of a cuvette through which water solutions could be flown. A glass prism was put in contact with the glass side of this wall. A He–Ne laser was used as the light source and a photodiode as a detector in a goniometer to measure the position of the resonance angle. It was found that the resonance angle increased with more than 20° going from air to water as the surrounding medium and that the resonance curve broadened, both in accordance with the theoretical models for SPR. Furthermore, the spontaneous adsorption of a protein (IgG) could be observed as well as the subsequent binding of its antibody (a-IgG). The resonance angle shift upon adsorption of IgG was 0.6°, which suggests an organic layer thickness of about 50 Å, a reasonable value for a monolayer of IgG. The binding of a-IgG caused a further shift of almost 1°. The analytical capabilities of the setup were elucidated using the initial kinetics of the resonance angle shift upon addition of the a-IgG. The angle of incidence was kept constant at the left part of the resonance curve with adsorbed IgG and the increase in light intensity upon addition of a-IgG measured for different a-IgG concentrations.

**Fig. 3:**
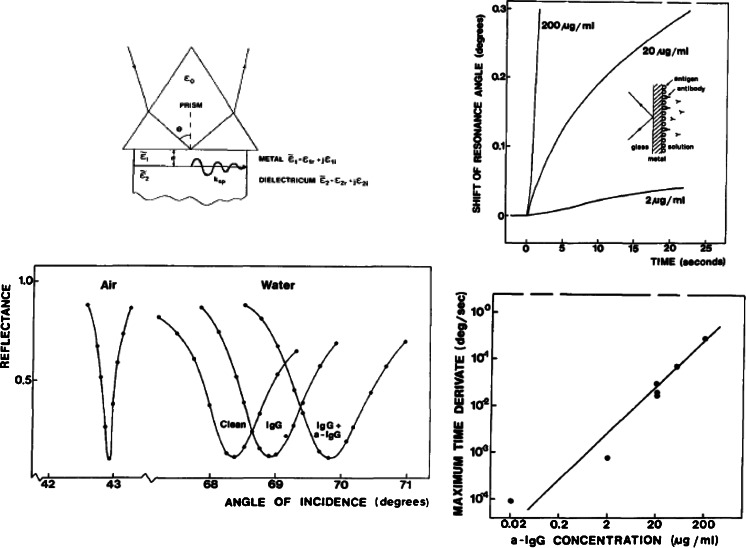
Summary of the first biosensing experiments with SPR. The change of the resonance angle with time (*upper right*) was calculated from the reflected light intensity change and the slope of the left part of the resonance curve. The maximum derivative of that change was observed to be approximately linearly related to the a-IgG concentration (Reprinted from ref. [1] with permission from Elsevier)

Since the antibody–antigen binding was irreversible, a new glass slide was used for each experiment. Our silver films of 60 nm thickness were, however, very reproducible and allowed the experiments above. The a-IgG concentration was inferred from the maximum slope of the transients and not from the initial slope since the manual introduction of the sample caused some uncertainties in the beginning. The results are summarized in Fig. 3. It was concluded that “we have demonstrated selective antibody reactions with a sensitivity that appears to be higher than in any other known physical method,” which was probably the case in 1983. In light of the developments occurring after the publication of the paper [1], it is interesting that we in its discussion section mentioned for example “by using hydrogels and Langmuir–Blodgett films, we hope to be able to construct a number of different types of SPR bioselective sensors.” Furthermore, it was stated that our experiments were made with silver but that gold may be superior due to its better stability in buffer solutions.

## Developments Making Surface Plasmon Resonance into a Pioneering Biosensor Technology

In the beginning of the 1980s, Pharmacia became interested in the possibilities given by biosensor technology to detect without labels the interaction between biomolecules. Discussions with the researchers at the Laboratory of Applied Physics regarding different alternatives were already ongoing at the time of the demonstration described above. A project was started in 1984 to further investigate the use of SPR for affinity-biosensing purposes. Researchers were employed both from the Laboratory of Applied Physics and from the Swedish Defense Research Institute in Umeå, where also studies of the interaction between biomolecules were made. Pharmacia Biosensor was formed in 1986 to develop, produce, and market an instrument for real-time biospecific interaction analysis without labels. The first products were launched in 1990, an instrument called BIAcore and a regenerable sensing chip onto which biomolecules could be coupled using known coupling chemistries. There will be other contributions in this issue dealing with the present day BIAcore instrumentation and its use. Here, we will summarize the initial improvements necessary to take the results presented in Fig. 3 to a commercially viable biosensing instrumentation, which today seems to be a “golden” standard for affinity-based measurements and often used in state-of-the-art biomedical research.

Some important developments are given in bullet form below:

- Regarding the sensing chip
  - Use of gold as the metal
  - Use of a dextran layer as the sensing matrix, including a self-assembled monolayer of alkane thiols to bind the dextran to the gold surface
  - Chemistry for covalent immobilization of ligands
  - Regeneration procedures making multiple use of the same chip possible
- Regarding the instrumentation
  - Cylindrical prism and fan-shaped light beam, resonance minimum detected as a dark band on a photodiode matrix or CCD (no moving parts)
  - Refractive index matching polymer (no immersion oil)
  - Temperature stabilized sample cell and optics
  - Thin sample cell and microfluidics allowing efficient and accurate delivery of biomolecules to the sensing chip

**Fig. 4:**
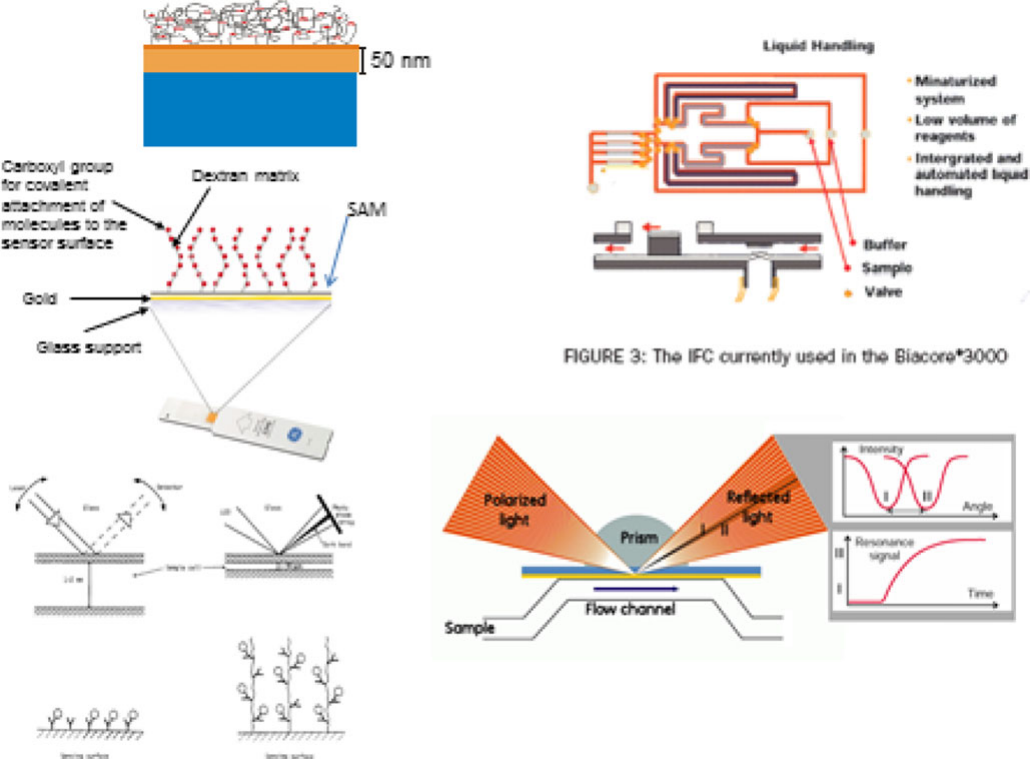
Schematic illustration of the differences between the first experiment and the developments made at Pharmacia Biosensor (Collage made from material obtained from Pharmacia Biosensor and ref. [18])

In addition to the points above, there were also developments of liquid handling (buffers, regeneration medium etc.), automatic sampling, and software for instrument control and for the elucidation of the generated kinetic-binding curves. Some of the points above will be schematically illustrated in the following. The bottom left-hand corner of Fig. 4 illustrates the main differences between the BIAcore and the original experiments. We used a sample cell with millimeter dimensions, whereas in the commercial instrument, the sample cell is much thinner, which gives a large laminar flow rate and efficient delivery of molecules to the sensing surface (smaller diffusion time constants). Furthermore, an extended sensing matrix can provide more detecting ligands per unit area than the surface itself. Since the evanescent light intensity outside the metal has a decay length of around 100 nm, a matrix of similar extension provides more sensitivity [18]. Another advantage is the fact that the dextran layer is mostly water, and therefore, the biomolecules will be present in a more native environment [19, 20]. The second big difference is in the optics, where a fan-shaped beam produces a dark band at a detector array at the surface plasmon resonance angle. The position of this band accurately calculated by evaluation software is now used as the signal caused by binding of molecules in the extended sensing matrix. Microfluidics with pneumatic-driven valves provides accurate sample injections and a lamellar flow above the sensing chip. A summary of the BIAcore technology is presented as a collage including sensing chip, microfluidics, and optics in Fig. 4.

There are many publications from 1990 and onward by researchers at Pharmacia Biosensor. A small number of these in addition to those already given are found as references [21–25]. Reference [23] is of particular interest. It contains 16 authors and it reflects, I believe, in a very clear way the multidisciplinary approach taken by Pharmacia in the development of biospecific interaction analysis. Reference [23], which in principle describes the complex new instrumentation, contains contributions from several disciplines like bio- and surface chemistry, optics, microfluidics, kinetic modeling, mechanical engineering, and software development. One of the early joint publications of Laboratory of Applied Physics and Pharmacia Biosensor is listed as reference [25].

Since the introduction of the BIAcore instrumentation in the beginning of the 1990s, there have been numerous applications regarding biospecific interaction analysis with the instruments but also a continuous improvement of the technology and its combination with other techniques. This is true not only for the BIAcore but also for the development of SPR for biosensing in general. These developments will most certainly be described in other parts of this issue and are not further elaborated upon here. It is in this context interesting to point at the overviews of optical biosensors published regularly by David Myszka and co-workers in *Journal of Molecular Recognition*. The last published survey at the time of writing covers the year 2009 [26].

**Fig. 5:**
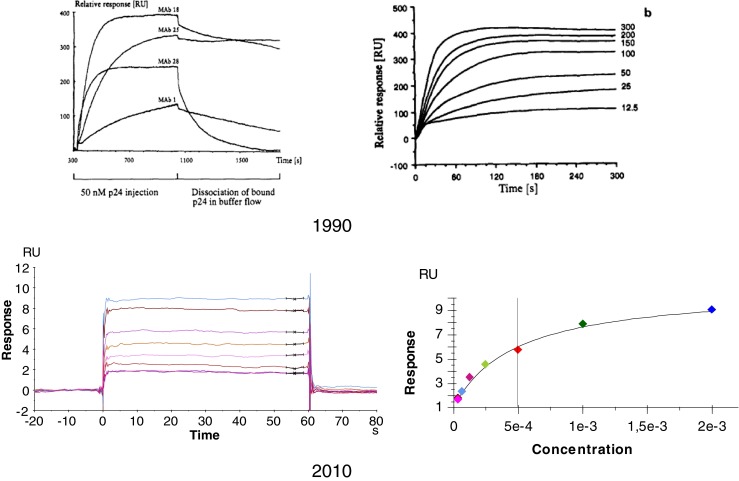
Illustration of the development of SPR for biospecific interaction analysis. *1990* Binding between antigens and antibodies. The example shows binding of p24 antigen to different monoclonal antibodies captured in the sensing matrix (left) and kinetics of binding to one of the antibodies (MAb 28) at different antigen concentrations (nM) (Reprinted from ref. [22] with permission from Elsevier). *2010* Kinetic analysis of small molecules possible with a resolution better than 1 Ru. The example shows methanesulfonamide (MW = 95 Da) binding to carbonic anhydrase immobilized in the sensing matrix (courtesy of Dr. Stefan Löfås, GE Healthcare)

The BIAcore development after the initial launch is exemplified by Fig. 5, where typical measurements at the time of launch (1990) and 20 years later are shown. It is now possible to obtain binding data for small molecules (MW ~ 100) compared to the binding experiments made mainly with antibodies (MW ~ 100,000) in the beginning. Figure 5 illustrates that the improved stability of the optical detection is one important factor in this development, allowing accurate measurements of a few RUs compared to tens of RUs from the start.

## Later Activities in Linköping

The pace of the development at Pharmacia Biosensor was very large and multidisciplinary in a way not possible at the university department. We were, however, collaborating with Pharmacia Bioenensor and initially also with the Defense Research Institute in Umeå on certain aspects of the SPR phenomenon and the surface chemistry of the sensing chips. The commercial development also put Linköping on the “biosensor map,” which led to several interesting international collaborations. The Laboratory of Applied Physics was one of the first university laboratories which got access to an early BIAcore instrument through a donation from Pharmacia Biosensor. The research in Linköping and the collaboration with Pharmacia were led and coordinated by Bo Liedberg after his PhD exam [27]. He and his research group (later the Division of Molecular and Surface Physics) have produced numerous interesting results regarding the development of SPR physics, technology, and applications. After the initial publication on SPR for biosensing, there have been several hundreds of papers and conference contributions published from Linköping in this area including studies of protein adsorption phenomena on solid surfaces also. The Laboratory of Applied Physics expanded during the 1990s and has given rise to several new professor chairs and consists today of nine different research divisions collected into a scientific area called “Applied Physics.” Numerous theses (~25) have been produced regarding different aspects of SPR, protein adsorption, surface modifications, and biospecific interaction analysis. The research from around 1980 to 2000 is exemplified by ten rather arbitrarily chosen references [28–37] in addition to the three papers already mentioned [9–11]. The last of these references introduces one of the larger research efforts by Bo Liedberg and his research group, namely imaging SPR [37–46]. A summary of the work on imaging SPR in Linköping and at other places up to 2010 is found in the review by Ekblad and Liedberg [45]. More recently localized SPR has been the included in the research projects of the group [47–53]. Several applied studies in collaboration with other research groups and other disciplines have been performed. One of the more unique applications is the use of imaging SPR to monitor the “touchdowns” of a marine microorganism on different types of modified gold surface and to correlate its colonization behavior with the amount of footprints left by the microorganism [43, 54].

## Computer Screen Photo-Assisted Techniques

**Fig. 6:**
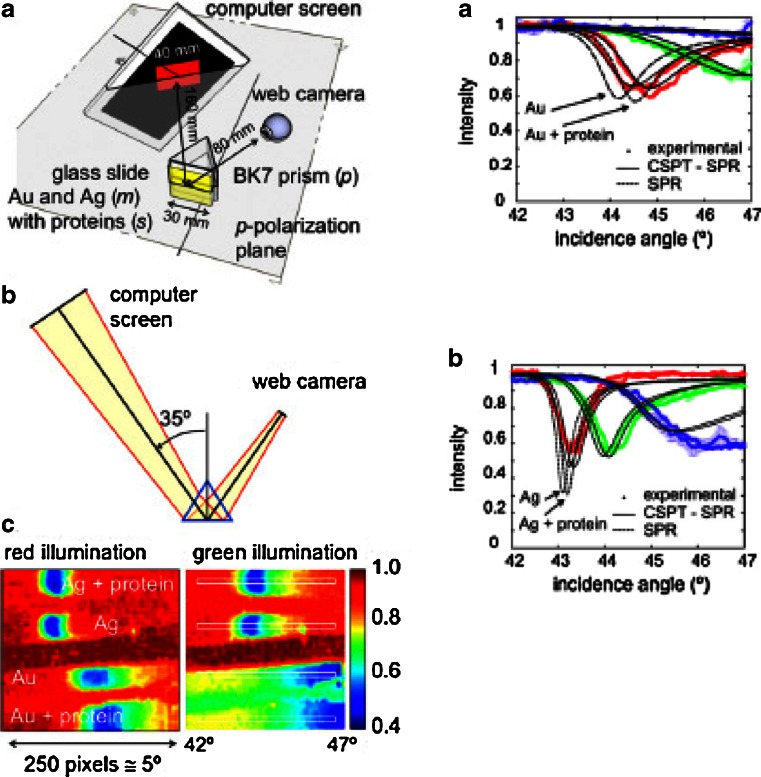
Illustration of the use of a computer screen as a light source and a web camera as a detector for SPR measurements of protein adsorption. Measurements performed in air. The *right-hand side diagrams* show the intensities of the light detected by the web camera at different angles of incidence for gold and silver for three different illuminations. The *black lines* show the calculated SPR resonances for monochromatic light and for the screen light (CSPT-SPR), respectively (Reprinted from ref. [59] with permission from Elsevier)

My own research interests from year 2000 onward have been partly in the use of ubiquitous instrumentation for bio- and chemical sensing, especially together with color indicators. The so-called computer screen photo-assisted technique (CSPT) was developed by Daniel Filippini [55]. It is based on the use of a (computer) screen as a light source and a web camera as a detector in optically-based assays. It was early used in collaboration with the University of Rome “Tor Vergata” to obtain optical fingerprints of the interaction between thin films containing color indicators, like metalloporphyrins, and gaseous species, which previously could be obtained only with rather advanced optical instrumentation [56]. This collaboration led among other things to an optically-based experimental model for artificial olfaction [57]. It was later demonstrated that CSPT could also be used for ellipsometry [58] and for SPR experiments [59]. The implementation of CSPT for SPR is illustrated in Fig. 6, where the polarized light of an LCD screen is used as the excitation source and a web camera as the detector. The middle left-hand image shows a ray tracing illustrating the image formation on the web camera. The bottom left-hand images are the color-coded light intensity images in the red camera channel for red illumination (rgb = 255, 0, 0) and in the green channel for green illumination (rgb = 0, 255, 0). The results for silver and gold sensing layers are given for the clean metal and for the metal with an adsorbed protein layer. As expected, the the SPR resonances are broad for the CSPT arrangement due to the broadband character of the illumination from the screen.

## SPR Biosensing with a Cell Phone

**Fig. 7:**
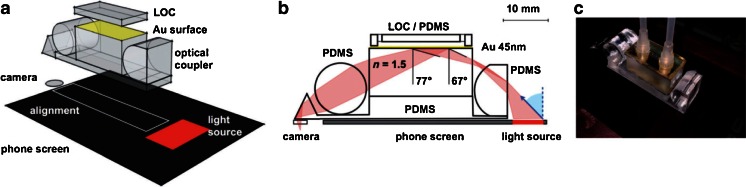
Illustration of angle-resolved SPR made with the illumination from the screen of a cell phone and the front camera of the phone as the imaging unit. The *middle drawing* shows the light path in the disposable optical coupler and the *right-hand image* is a photo of the actual experimental device (Reprinted from ref. [60] with permission from Wiley-VCH Verlag GmbH & Co)

**Fig. 8:**
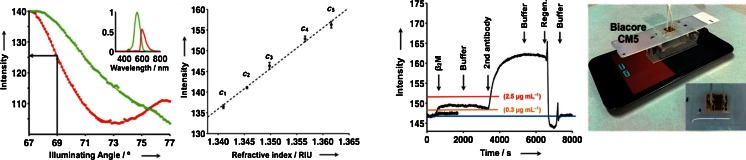
Angle-resolved SPR image using with the setup in Fig. 7. The 10° dip for red illumination corresponds to 90 pixels in the image. The *second drawing from the left* is a calibration curve where the intensity at a given angle (69°) is measured for solutions with different refractive indices. The *two drawings to the right* illustrate the detection of β_2_-microglobulin with a BIAcore sensing chip. See ref. [60] for details (Reprinted from ref. [60] with permission from Wiley-VCH Verlag GmbH & Co)

The further development of the use of ubiquitous devices for biosensing purposes made by Filippini and his co-workers get a separate section since it connects his activities very well with the topic of the present contribution. They have recently shown that with a designed sample cell and optics, it is possible to make SPR detection also with a cell phone. Most interestingly, it is possible to design the sample cell, so it can be used with a BIAcore sensing chip for (kinetic) evaluation of biomolecular interactions [60]. The sample cell, experimental setup, and results related to biosensing are shown in Figs. 7 and 8. It is concluded that β_2_-microglobulin can be detected in the clinically interesting range with the setup in Fig. 7. The development included not only the design of an optocoupler and a lab-on-a-chip layout making SPR with the cell phone possible but also the development of the necessary software to master the acquisition of the data from the image obtained by the front camera. The kinetic-binding curves shown in Fig. 8 were obtained as the light intensity at a given angle of incidence. According to the authors, the sensitivity and the resolution of the cell phone-based SPR sensing compare well with that of other compact SPR devices.

## Conclusions

“Is SPR the best physical principle for affinity-based biosensing without labels?” This is a question which has been often asked during the (30) years. Efficient and stable SPR optics together with an optimized sensing matrix with low unspecific adsorption and an efficient immobilization of ligands and a sample cell providing a well-defined lamellar flow give a very good overall performance of SPR instrumentation. The issue is if any other surface-oriented physical detection method could provide a better performance together with a sensing matrix and efficient sample handling. Quartz crystal microbalances seem here to be interesting alternatives, although quantitative results may be more difficult to obtain due to the interference of water associated with the biomolecular binding pair. An advantage of SPR may also be that the sensing chip is produced separately and allows a hydrogel as the sensing matrix. The sensing chip is not a part of the detection device itself, and it is relatively simple to make multichannel or multisport assays for imaging applications. Improved sensitivity may come from the use of specific measurement modes, like “SPR-ellipsometry,” i.e., utilizing changes in the phase shift of the light at the surface plasmon resonance angle. Breakthroughs are likely to come from the numerous demonstrations of localized surface plasmon resonance (LSPR). Biosensing with cell phones using standard sensing chips is another interesting possibility, which allows SPR-based evaluations to be performed with a widely distributed instrument, namely the cell or smart phone with a camera in its front. The lab-on-a-chip layout providing assays with commercially available sensing chips make the approach of interest in a number of diagnostic situations in a point-of-care setting.
